# Paediatric Over-the-Counter (OTC) Oral Liquids Can Soften and Erode Enamel

**DOI:** 10.3390/dj5020017

**Published:** 2017-05-11

**Authors:** Dan Zhao, James Kit-Hon Tsoi, Hai Ming Wong, Chun Hung Chu, Jukka P. Matinlinna

**Affiliations:** 1 Dental Materials Science, Applied Oral Sciences, Faculty of Dentistry, the University of Hong Kong, Hong Kong SAR, China; hannahziu@163.com (D.Z.); jpmat@hku.hk (J.P.M.); 2 School of Stomatology, Zhejiang Chinese Medical University, Hangzhou 310053, Zhejiang, China; 3 Paediatric Dentistry, Faculty of Dentistry, the University of Hong Kong, Hong Kong SAR, China; wonghmg@hku.hk; 4 Operative Dentistry, Faculty of Dentistry, the University of Hong Kong, Hong Kong SAR, China; chchu@hku.hk

**Keywords:** OTC drugs, paediatrics, enamel, hardness, pH, oral liquids

## Abstract

This study investigated the softening and erosive effects of various paediatric over-the-counter (OTC) oral liquids on deciduous teeth. Twenty sectioned and polished deciduous enamel blocks were ground on the buccal surface (2 × 2 mm^2^) and randomly divided into five groups, immersed into four commercially-available paediatric OTC oral liquids (two for paracetamol, both sugared; and two for chlorpheniramine, one sugared and one sugar-free), with deionized water as control. The pH of the oral liquids ranged from 2.50 to 5.77. Each block was immersed into the test or control groups for 15 s, rinsed with deionized water, and Vickers micro-hardness (*n* = 5) was measured. After twenty cycles of immersion and hardness measurements, Scanning Electron Microscope (SEM) and Energy Dispersive X-ray Spectrometry (EDS) were used to evaluate the surface morphology and chemistry of the tooth blocks, respectively. The pH values of the liquids were also recorded. Rapidly descending trends in the micro-hardness ratios of the four test groups were observed that were statistically different from the control group (*p* < 0.001). EDS showed an increase of Ca/C ratio after drug immersion, whereas SEM showed an enamel loss in all the test groups. Paediatric OTC oral liquids could significantly soften the enamel and render them more susceptible to caries, such that the formulation of the oral liquids is the major factor.

## 1. Introduction

Dental erosion is defined as the “irreversible loss of tooth structure due to chemical dissolution by acids without the involvement of bacteria” [1], and may combine with mechanical activities such as abrasion and attrition [2]. The sources could be intrinsic or extrinsic. Intrinsic sources include acid reflux, emesis or regurgitation due to some gastrointestinal diseases; on the other hand, extrinsic sources of acids are derived from acidic foods, beverages such as sport drinks and wines, and acidic medications [3].

Erosion begins on the enamel surfaces and then proceeds to the underlying dentine if no timely intervention is instituted. In brief, the initial stage is softening of enamel surface and the degree varies with the immersion time and the type of acids involved. Subsequently, if the erosive attack continues, dissolution of enamel crystals takes place, which is a permanent loss with a rough layer on top of the remaining tissue [4]. Indeed, in a chemical sense, there is a “critical pH of enamel”, which is defined as the pH at which a solution is just saturated with respect to mineral of enamel, and the enamel on tooth surface will be in equilibrium with no dissolution or mineral precipitation occurring [5]. That means below the critical pH, the solution is going to be under-saturated and the potential for enamel dissolution exists. 

Epidemiological surveys about dental erosion in preschool children have been conducted worldwide. The occurrence of dental erosion indeed varies from 25% to 75% due to the different factors including but not limited to countries, dietary habits and the lack of a standardised diagnosis guideline. For example, one survey [6] in Hong Kong was conducted to assess the prevalence of erosion among 12-year-old children in seven primary schools, and it was found that most children (75%) had at least some sign of erosion. It may thus be inferred that the occurrence of dental erosion in children is quite high, which should raise a concern among paedodontists. 

With a considerably increasing intake of beverages such as soft drinks, energy drinks and soda, which contain a high concentration of fermentable carbohydrates [7], dental erosion among children becomes a public health concern due to its high prevalence [6,8,9]. Clinically, the early stages of erosion are often overlooked by the children themselves and doctors, possibly due to a lack of perceivable clinical signs and symptoms [10]. Thus, if there is no timely intervention, children may suffer from severe enamel surface loss, tooth sensitivity, poor aesthetics or even dental pulpitis, which will necessitate complicated treatment and lead to a compromised dentition for the entire life [10]. While in some cases dental erosion can be controlled through modification of the children’s dietary habits, other children may be on long term medication which predisposes them to dental erosion, in particular those who are suffering from chronic diseases like respiratory allergies, asthma, cardiopathy and epilepsy, and it is in these cases where the possible incidence of dental erosion cannot be neglected [11].

As a matter of fact, some drugs, e.g., respiratory drugs, are commonly prescribed to children before two years of age [9]. Although many solid forms of oral medication such as pills or capsules have a coating to mask their bitter tastes, such methods are impractical for many children since the children are too young to swallow the pills and capsules. The situation varies greatly among older children [12]. Consequently, the most common choice of formulations for children are in liquid form. One of the biggest challenges of administering medicine in children is a “matter of taste”, as drugs often taste bitter due to their chemical nature. In order to minimise the unpleasant taste of bitterness, paediatric medications are usually coloured, flavoured and sweetened with various excipients besides containing the main active ingredients. These additives include bulk materials (such as for thickening), flavourings, sweeteners, buffers, acids, preservatives and colouring agents, which are prevalent among formulations for children [12]. The frequent use of acids in paediatric medicine is associated with an improvement in flavour [13,14]. Meanwhile, proper liquid formulations have high requirements regarding the maintenance of solubility, chemical stability, taste masking and preservation. For liquid drugs, a suitable pH is needed at which the drug is both sufficiently soluble and chemically stable. Acidic contents are added into formulations as buffering agents, and are responsible for controlling tonicity and ensuring the drugs’ physiological compatibility [13].

However, frequent use of syrups sweetened with sucrose or fructose or with a combination of both has been linked to dental caries and a drop of plaque pH in the long term [15,16,17,18,19,20], and the cariogenic potential of paediatric liquid medicines is probably the result of a high concentration of fermentable carbohydrates and their acidogenicity [21]. It has been reported in one study that about half of 97 regularly-used paediatric medication formulations have an endogenous pH below 5.5 and thus are capable of damaging tooth enamel [13]. In addition, consumption of such drugs at night could aggravate the dental caries and dental erosion, because during this period the salivary flow rate is reduced and the time of elimination from the oral cavity increases [22]. 

Nowadays, pharmaceutical companies recommend using sugar-free medicines that are oral liquid formulations without fructose, glucose, or sucrose. It seems to be a plausible option to replace the sugar-containing medications, but there is still no consensus as to whether they have true benefits or not. According to one report, it was found that the replacement sugar-free medicines might themselves damage teeth, which was not desirable. In fact, a reduction of the sugary ingredients in the medicines might entail the addition of weak acids to make up for their palatability concerns and optimise formulation properties, which would bring up dental erosion [20]. A comprehensive review [23] by Strickley et al. has illustrated various functions of the excipients in paediatric drug formulation, whilst a recent report [24] has expressed a very great concern about the potential risk of drug-induced caries from sugar or its carbohydrates and their negative effects on the oral health (and the future growth of adult dentition) of children. However, data about erosive potential and enamel softening of commonly-used over-the-counter (OTC) paediatric oral liquids is lacking.

The aim of this study was to evaluate the erosive effect of the paediatric oral liquid medicines on deciduous teeth. The null hypothesis for the study was that there was no difference of enamel between the test and control groups after immersion challenge, which means the liquids would not cause erosive damage.

## 2. Results

### 2.1. pH Values

Table 1 shows the pH value of the test groups. The pH values ranged from 2.50 (group GC) to 7.17 (group GE) with similar endogenous pH among paracetamol groups and only one medication that was slightly higher than 5.5 (group GD). These measurements indicate that all the tested paediatric OTC medications exhibited an acidic pH.

### 2.2. Surface Micro-Hardness

**Figure 1:**
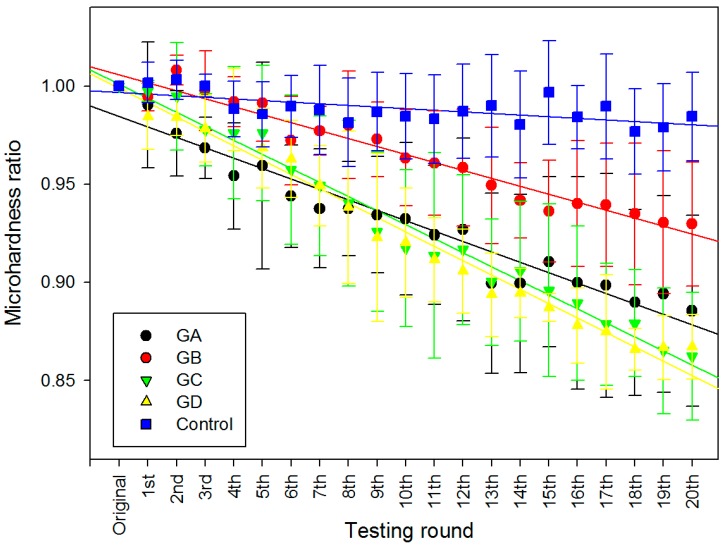
The relationship between mean micro-hardness ratio and standard deviation vs. testing rounds. GA and GB groups contain paracetamol, GC and GD groups contain chlorpheniramine, GE is the control group which is deionised water.

The mean micro-hardness ratios (MHR) in each group, as a function of testing round k, are shown in Figure 1. In general, the MHR decreased with the number of drug immersion in all experimental groups despite variations in the magnitude. In the control group, only a small fluctuation (<6%) on MHR was observed after 20 rounds of immersion. However, there were drastic decreases of around 15% in the MHR in both the GC and GD groups, whilst the GB group only exhibited a 7% decrease in MHR. One-way ANCOVA revealed that test groups collectively have statistically significant differences from one another (Table 2), and the control group has a statistically significantly different regression slope from all others (*p* < 0.001), whilst GA, GC and GD has no statistical significant difference between each other (*p* > 0.05) (Table 3).

### 2.3. Scanning Electron Microscope (SEM) / Energy Dispersive X-Ray Spectrometry (EDS) Analysis

**Figure 2:**
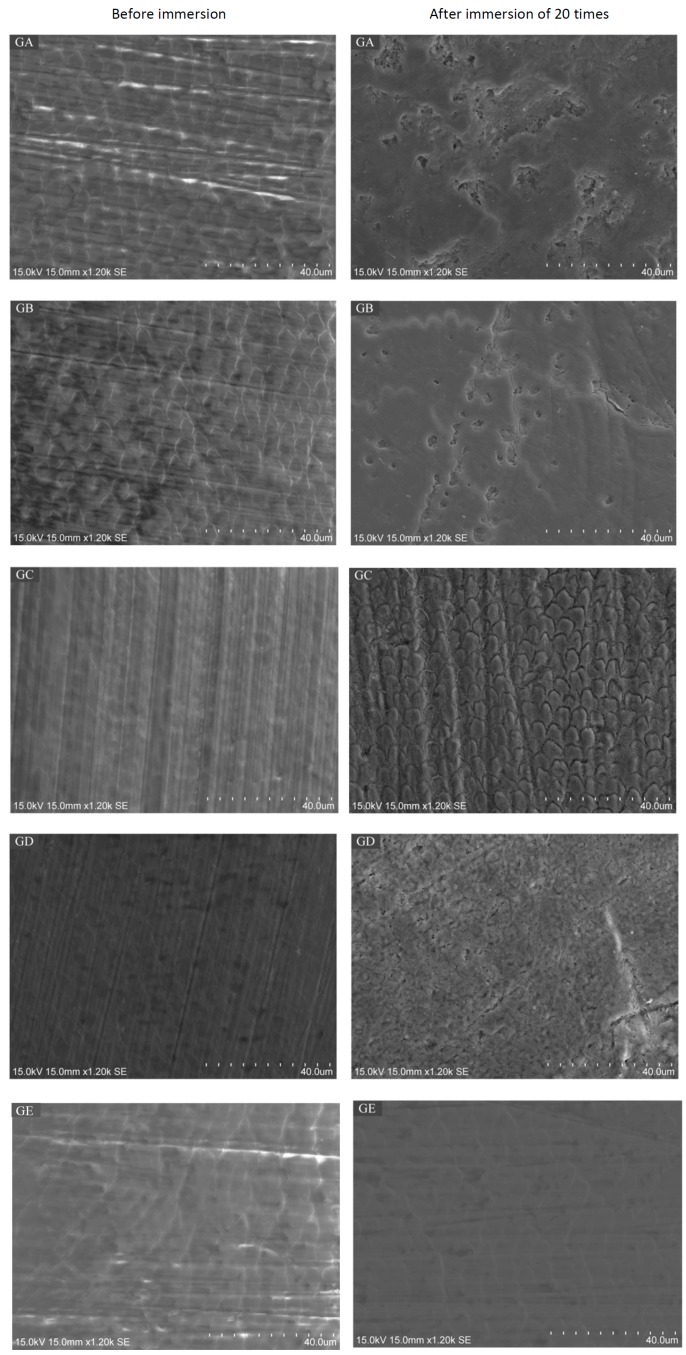
Scanning electron microscope (SEM) micrographs (×1200) of deciduous enamel before and after immersion 20 times in drugs containing paracetamol (groups GA and GB), chlorpheniramine (groups GC and GD), or deionized water (Control group).

The median values for the three repeated EDS measurements in each sample before and after the experiment were calculated and analysed in the Kruskal–Wallis test (Table 4). Generally, the Ca/C ratio decreased after the drug immersion, while the Ca/P ratio and Mg, Na weight percentages mostly remained stable. However, no statistically significant differences have been found. Moreover, the data revealed that, before the immersion, all enamel surfaces appeared to be flat (Figure 2). After the twenty rounds of immersion, the surfaces of the GA group displayed a distinctive enamel loss with irregular craters. The surfaces of the GB group showed a corroded surface and the fracture lines were along the border of prism heads. In group GC, the surfaces presented a remarkable acid-etched prism-sheath structure of enamel, with the prisms surrounded by a wide sheath region. Group GD also exhibited disorganised enamel loss with increased porosity on the surface. No distinctive microstructure loss was observed in control group (group GE).

## 3. Discussion

In this in vitro study, we found that the OTC paediatric oral liquids could significantly lower the Vickers micro-hardness of enamel, i.e., had a softening effect, causing damage on the surface of enamel as seen from SEM after successive immersion cycles. It seems that the enamel of deciduous teeth is susceptible to acid erosion caused by the oral liquids, while the presence of sugar or pH of the oral liquids were not the major factors. 

Three of the tested medicines showed a pH lower than the “critical pH of enamel”, i.e., 5.5. However, impurities in the dental mineral may increase its solubility, thus the relative rates of dissolution do not truly reflect the solubility [25]. It was difficult to evaluate the solubility products based on the actual mineral composition, since it has been demonstrated that the minerals in biological samples were heterogeneous [26]. Accordingly, the critical pH for enamel was typically considered to be in a range of 4.5 to 5.5 [27]. Even so, the current study confirmed that liquid medication with an endogenous pH of 5.7 might also cause erosive damage to the enamel surface. In fact, the rate of dissolution of enamel is strongly influenced by other physical factors (e.g., temperature, consumption frequency) and biological factors (e.g., saliva in the formation of the acquired pellicle, as a lubricant, buffer and ion reservoir). For example, solubility is dependent on temperature and heat is required to break the bonds holding the molecules in a solid [28]. Thus, erosion is predicted to be more severe at high temperatures and less at low temperatures, which has been shown by Banan et al. that dairy beverages caused less decrease of pH in plaque and saliva at temperatures lower than room temperature [29,30].

In addition, from a biological perspective, the presence of saliva is greatly beneficial to an oral cavity under erosive attack. Saliva is required to form the acquired pellicle, which is defined as a proteinaceous layer on all solid surfaces exposed to the oral cavity [31,32,33]. It is an organic film composed of glycoproteins and proteins, including several enzymes, without the involvement of bacteria [31,32]. It works as a protective membrane to prevent direct attack from acidic substances, thus lowering the rate of demineralisation [33]. Furthermore, bicarbonate is the most important component in saliva to protect against acidic products generated by dental plaque or extrinsic acids [34]. The presence of calcium and phosphate, as well as an alkaline or neutral environment is essential for remineralisation. Preserving calcium and phosphate in saliva allows them to penetrate into the minerals of enamel, slowing down the rate of mineral dissolution [35]. One research study has shown that salivary proteins would bind to demineralised sites and cover exposed crystals by a specific adsorption mechanism [36]. In the oral cavity, the contact of the enamel with acidic substances is usually only transient before clearance by saliva. Consequently, under in vivo conditions, an early stage of erosion is limited to a very insignificant loss of mineral and erosive craters on a nanometer scale or even near atomic level [37]. In this sense, there might be discrepancies between the current in vitro study and other in vivo studies.

Since there was no involvement of saliva in this study, it is possible that the erosive potential of medications was overestimated. For example, we have not included the buffer capacity of saliva which could alter the final pH in the oral cavity and hence lower the dissolution rate of enamel [38], possibly due to the high bicarbonate concentration in saliva [39]. Nevertheless, significantly different buffering capacities have been found between genders (e.g., male and female), as well as the method of collection (e.g., unstimulated or stimulated) [38]. Thus, in most of the in vitro studies, various formulations of artificial saliva were employed. However, most simple simulated saliva compositions have no proteins and do not form acquired pellicles, which play an important protective role against erosion. Moreover, collection of natural saliva in a restricted time frame is not a simple task, particularly with the quick deterioration of saliva [40]. Therefore, more in vivo studies are necessary to reliably evaluate the erosive effect under the influence of saliva and masticatory activities.

Deciduous tooth enamel is softer than permanent tooth enamel. A study has found that deciduous teeth had a lower prismatic density, which meant a smaller number of prisms per unit area, and a shorter prismatic diameter compared to permanent teeth despite having the same morphology [41]. The higher percentage of minerals makes the enamel more resistant to masticatory forces, but its extreme hardness makes it more prone to fractures and more reliant on the presence of the dentine—which is more resilient—to maintain its integrity [42]. Nevertheless, some investigators found that deciduous enamel was softer and more prone to fracture than permanent enamel [43]. Furthermore, the lessened mineralisation and the smaller thickness in deciduous enamel make primary teeth more prone to erosion and decay [44,45,46]. Generally speaking, the erosive potential of primary enamel is greater and its weaker mechanical properties could be explained by the reduced mineralisation in deciduous enamel tissue.

In the current study, the deciduous teeth were challenged by 20 rounds of 15 s drug immersion time. Fifteen seconds was chosen for the immersion time so as to reflect the drug taking habits of children and the residence time of drugs on enamel. The twenty rounds were to mimic the times of consumption (*quater die sumendus* for five days). In addition, since the initial values of micro-hardness differed among the specimens, ratios (MHR) were calculated instead and corresponding trend lines were drawn in order to allow for better data comparison among the groups.

Liquid formulations are usually complex compositions containing many other components besides the main active ingredients; the additives include bulk materials, flavourings, sweeteners, buffers, preservatives and colouring agents [12]. Although we do not know the exact formulation of the purchased medicinal liquids, acidic contents were common to all the liquids and acted as buffering agents, and they were responsible for improving palatability, maintaining chemical stability, controlling tonicity and ensuring the physiological compatibility [13]. For example, citric acid was the most frequently added acid, yet it has been linked to tooth erosion due to its ability to dissolve the hydroxyapatite of tooth enamel and dentine [47]. It has been found that citric acid acts as a chelator that binds to the minerals of the hydroxyapatite, reduces saliva supersaturation and increases the dissolution rate of hydroxyapatite crystals [48]. Other acids have also demonstrated various erosive effects on enamel; it has been reported that lactic acid at low pH was more erosive than both citric and maleic acids [49]. 

In the chlorpheniramine groups, a sugar-free formulation group GD having a higher pH value exhibited a similar micro-hardness decreasing trend as group GC. It might be due to the addition of “sugar-free” excipients, i.e. artificial sweeteners, are acidic or chemically stable under acidic environment [50]. This result was further confirmed by another study of comparing the erosive potential between sugar-free medicine and sugar-containing ones. They found that the sugar-free medications still carried a similar erosive potential as the sugar-containing syrups [13]. In addition, the active ingredient of these two drugs was chlorpheniramine maleate, which in fact would be dissociated into chlorpheniramine free base and maleic acid in aqueous conditions [51]. Indeed, despite maleic acid being a weak acid, it has the potential to etch [52] or chemisorb [53] on enamel, so much so that it could be utilised for bonding orthodontic brackets onto enamel [54]. It seems to be that simply shifting to sugar-free medications is not as beneficial as once thought, and a fundamental understanding of the formulation science and chemistry is essential. 

With respect to the elemental analysis by EDS, semi-quantitative data were presented to show the chemical changes of enamel surface after liquid medicine immersion. A previous study has examined the Ca/P and Ca/C ratio to compare the elemental difference before and after demineralisation, since it was thought that the ratios were altered from calcium hydroxyapatite due to the alteration or substitution of mineral phase [55]. However, in the present study, the Ca/P ratio before drug immersion determined by using the EDS was found to be around 1.9, which was not significantly different from that of enamel which experienced drug immersion. Accordingly, we speculate that the components of Ca and P change together (i.e., dissolved in the oral liquids) in the same direction. Besides, several other studies were unable to demonstrate any significant difference in the Ca/P ratio in compromised enamel [55,56]. Multiple studies on developing enamel have shown that the Ca/P ratio was constant during tooth development despite variations among individuals [57,58]. Nevertheless, Jälevik and co-workers did find that median Ca/P ratio of hypo-mineralised enamel was significantly lower than sound enamel [59]. Therefore, the teeth collected in various stages in different countries might have high variations which eventually led to the scattering of results. 

One study also revealed a relation between micro-hardness and the Ca/C ratio in demineralised enamel [56]. As the crystals were dissolved with more organic components exposed, it seemed plausible that the Ca/C ratio would increase. However, in this study, despite a Ca/C ratio decrease after drug challenge, the difference had no statistical significance. The limited sample size in this study and variations among individuals could contribute to the contrasting result. 

Given the findings of this study, paediatricians and patients should realise the risk of erosion during the consumption of liquid medicines by children. Dentists should not overlook the early stages of erosion, regarding minor tooth surface loss as a normal occurrence of daily life, since early diagnosis and appropriate intervention are seldom reached in time [60]. Children should also visit dentists regularly and oral hygiene education in preschool is necessary. Other methods such as an adequate use of fluoride-containing mouth-rinses, toothpastes, tablets and lozenges have been demonstrated to remineralise enamel effectively [61]. In future, more clinical studies or in vivo studies are needed, since the limits of in vitro studies do not allow simulation of all the complex biological effects. We anticipate this study could raise awareness of the erosion susceptibility from paediatric OTC oral liquids. 

## 4. Materials and Methods 

**Figure 3:**
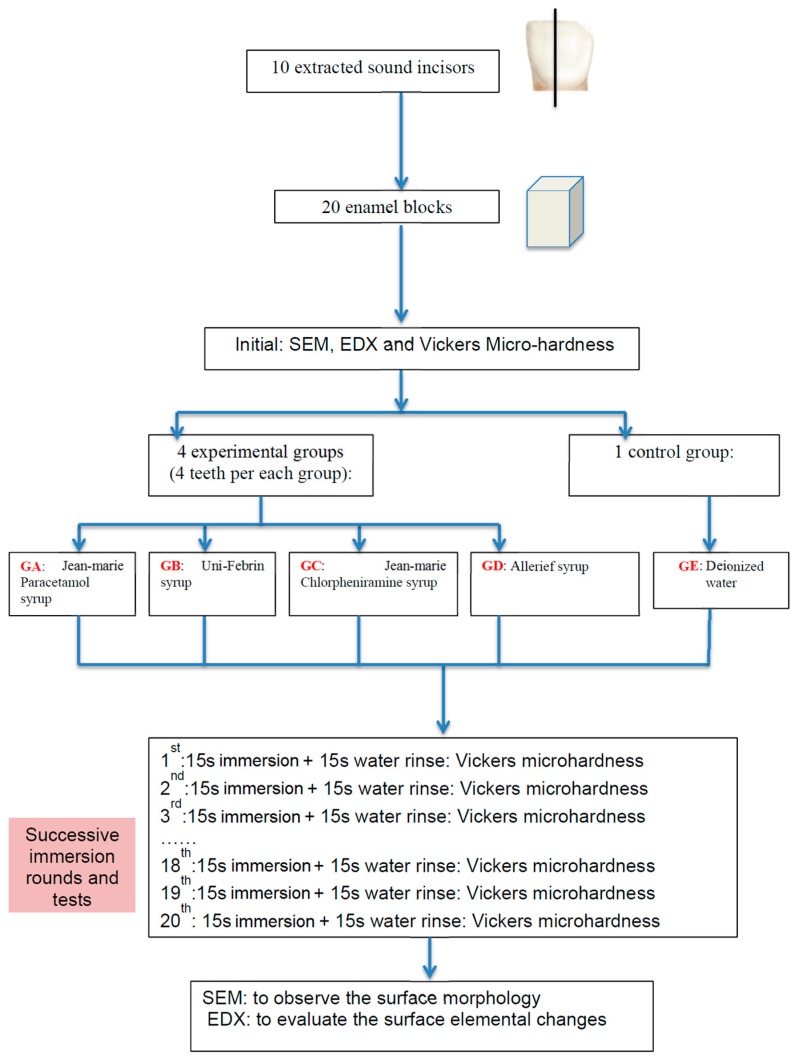
The flowchart of experimental design.

This study was approved by the Institutional Review Board of Hong Kong (IRB UW15-172). The procedure has been illustrated as a flowchart in Figure 3. 

### 4.1. Teeth and Specimen Preparation

Twenty sectioned enamel blocks, with 2 × 2 mm^2^ buccal enamel surface, were obtained from ten sound extracted primary incisors. The incisors were stored in distilled water at 4 °C prior to use for no longer than one week. Each incisor was sliced longitudinally parallel to its long axis to get two enamel blocks by a cutting machine under running water. All enamel blocks were embedded in light-cured resin (Filtek Z250, 3M ESPE) onto an acrylic plate. The buccal surfaces were faced upwards to make sure most regions of the surface are parallel to the plate. 

After embedding the enamel into the resin, the buccal surfaces of the enamel were polished using 1000- and 2000-grit SiC abrasive paper by a manually water-cooled low-speed polishing machine to obtain a flat surface without dentine exposure, and the specimen underwent ultrasonic washing in deionised water before and after polishing. Then, the twenty blocks were randomly divided into five groups (four experimental groups GA, GB, GC, GD, and one control group GE; see Table 1), i.e., four blocks in one group, to immerse into the oral liquids or deionised water.

### 4.2. Oral Liquids

As the most commonly used paediatric OTC drugs to relieve respiratory symptoms were usually for acute occasions [9], two commonly-used single component formulations, i.e., paracetamol and chlorpheniramine, were used (Table 1). Paracetamol (in US: acetaminophen) is a major ingredient in numerous cold and flu remedies and it is also classified as a mild analgesic to relieve headaches, fever and other minor aches and pains. Chlorpheniramine is an antihistamine used to relieve the symptoms of allergic conditions and the common cold, such as rhinorrhoea. Two different brands of paediatric OTC oral liquids containing one of each of the drugs were selected to make a comparison. In the chlorpheniramine group, the Jean-Marie brand contains sugar, while the Allerief belongs to a sugar-free type. All of the OTC drugs were purchased from local pharmacies and no further information from manufacturers was acquired. 

### 4.3. pH Measurement 

The endogenous pH of the testing medications was determined at room temperature by using a digital pH meter (CyberScan pH500, Eutech Instruments, UK) in triplicate to get a mean value. The pH meter was calibrated before measurement according to the manufacturer’s instructions, using buffer standards of pH 4 and pH 7. Then, 10 mL of the testing medicine was placed in one beaker, the probe of the pH meter was immersed directly into the syrup and the value was recorded with the precision of 0.01.

### 4.4. Micro-Hardness Measurement

To evaluate and compare the erosive potentials of four medications on a continuous scale, each specimen was subjected to twenty rounds of immersion in one of the drugs, followed by micro-hardness measurements. To set a baseline of the mean initial micro-hardness (MH_0_), each enamel block was immersed into deionised water (DI) for 15 s, and then five random spots were picked and tested using a micro-hardness tester (Leica DC 300, Leitz, Germany) with a Vickers tip and load of 200 g (1.962 N) lasting for 15 s. Then, in each round *k*, the enamel block was immersed into 10 mL of the corresponding test group for 15 s and followed by rinsing with DI water for 15 s. Again, the average micro-hardness values (MH*_k_*) of the enamel surfaces were measured immediately from five random spots using the same micro-hardness tester and parameters. The same immersion and measuring procedures were repeated for twenty rounds (i.e., *k* = 1–20). Fresh drug or DI water was used for each new round of immersion. As a whole, each sample experienced 21 (one initial + twenty after drug immersion) micro-hardness measurements. All the values were then plotted as the ratio (MHR) vs. round of immersion to give a trend (Figure 1) on how the surface hardness changes following a continuous drug attack, i.e.,:

MHR = MH*_k_*/MH_0_ (1)

where MH*_k_* is the mean micro-hardness at *k* = 0–20 rounds, and MH_0_ is the mean initial micro-hardness. The MHR and slope of regression lines of Figure 1 was statistically analysed by one-way ANCOVA at α = 0.05 (SPSS v.22, IBM, New York, NY, USA).

### 4.5. Scanning Electron Microscope Analysis

The surface topography of the enamel specimens at 1200× magnification was studied under a scanning electron microscope (Hitachi SU-1510 Variable Pressure SEM, Hitachi Ltd., Tokyo, Japan) at 15 kV in high-vacuum mode. The SEM micrographs of all enamel blocks were taken on two occasions: (1) before the immersion procedures, where the enamel blocks were mounted onto an aluminium stub separately to assess their surface topography without coating, and (2) after 20 rounds of immersion, where all the specimens were sputter-coated with carbon at 15 kV and the entire buccal surface was scanned. The SEM micrographs provided visual and illustrative comparisons of the surface morphologic changes of specimens before and after the drug/water challenge. 

### 4.6. Energy Dispersive X-Ray Spectrometry Analysis

The same samples that had been observed in SEM were also used for the EDS analysis on the same occasions, except the EDS was done prior to the coating. The EDS system comprised of an X-ray detector system (Model 550i, IXRF-Systems, Austin, TX, USA) attached to the SEM operated at 15 kV with a spot size of 5 nm, specimen tilt of 35°, working distance of 15 mm and a counting time of 20 s. Three counts from each enamel sample were measured to give a mean weight percentage of calcium (Ca), phosphorus (P), sodium (Na), magnesium (Mg), carbon (C) and oxygen (O). The mean wt % ratios of Ca/P and Ca/C, and the wt % of Mg and Na before and after tests were statistically evaluated by Kruskal–Wallis test at α = 0.05 (SPSS v.22).

## 5. Conclusions

Within the limitations of this laboratory study, the four tested paediatric OTC oral liquids were found to significantly soften enamel of primary teeth and deem them susceptible to caries. There is a possible association between drug formulations and their erosive potentials.

## Figures and Tables

**Table 1:** The information of test medicines and their pH in this study.

Test Group	Main Component and Concentration	Brand	Manufacturer of Drugs	pH (± SD); (*n* = 3)
GA	Paracetamol (120 mg per 5 mL)	Jean-Marie Paracetamol syrup	Jean-Marie Pharmcal, Hong Kong	4.97 ± 0.01
GB		Uni-Febrin syrup	Universal Pharm, Hong Kong	4.74 ± 0.01
GC	Chlorpheniramine (2 mg per 5 mL)	Jean-Marie Chlorpheniramine syrup	Jean-Marie Pharmcal, Hong Kong	2.50 ± 0.01
GD		Allerief syrup	Percuro Medica Ltd, UK	5.77 ± 0.01
GE	Control group	Deionised water		7.17 ± 0.06

**Table 2:** One-way ANCOVA results of micro-hardness ratios (MHR).

Dependent Variable: MHR								
Source	Type III Sum of Squares	df	Mean Square	F	Sig.	Partial Eta Squared	Noncent. Parameter	Observed Power ^b^
Corrected Model	0.158 ^a^	5	0.032	119.792	0.000	0.858	598.961	1.000
Intercept	27.991	1	27.991	106,059.503	0.000	0.999	106,059.503	1.000
Round	0.093	1	0.093	354.279	0.000	0.782	354.279	1.000
Drug Type	0.064	4	0.016	60.926	0.000	0.711	243.706	1.000
Error	0.026	99	0.000					
Total	94.592	105						
Corrected Total	0.184	104						

^a^ R Squared = 0.858 (Adjusted R Squared = 0.851); ^b^ Computed using alpha = 0.05.

**Table 3:** One-way ANCOVA pairwise comparisons.

Dependent Variable: MHR						
(I) Drug Type	(J) Drug Type	Mean Difference (I–J)	Std. Error	Sig. ^b^	95% Confidence Interval for Difference ^b^	
					Lower Bound	Upper Bound
GA	GB	−0.033 *	0.005	0.000	−0.043	−0.023
	GC	0.004	0.005	0.422	−0.006	0.014
	GD	0.007	0.005	0.180	−0.003	0.017
	GE	−0.056 *	0.005	0.000	−0.066	−0.046
GB	GA	0.033 *	0.005	0.000	0.023	0.043
	GC	0.037 *	0.005	0.000	0.027	0.047
	GD	0.039 *	0.005	0.000	0.030	0.049
	GE	−0.023 *	0.005	0.000	−0.033	−0.013
GC	GA	−0.004	0.005	0.422	−0.014	0.006
	GB	−0.037 *	0.005	0.000	−0.047	−0.027
	GD	0.003	0.005	0.604	−0.007	0.013
	GE	−0.060 *	0.005	0.000	−0.070	−0.050
GD	GA	−0.007	0.005	0.180	−0.017	0.003
	GB	−0.039 *	0.005	0.000	−0.049	−0.030
	GC	−0.003	0.005	0.604	−0.013	0.007
	GE	−0.063 *	0.005	0.000	−0.073	−0.053
GE (Control)	GA	0.056 *	0.005	0.000	0.046	0.066
	GB	0.023 *	0.005	0.000	0.013	0.033
	GC	0.060 *	0.005	0.000	0.050	0.070
	GD	0.063 *	0.005	0.000	0.053	0.073
Based on estimated marginal means						

*. The mean difference is significant at the 0.05 level; ^b^ Adjustment for multiple comparisons: Least Significant Difference (equivalent to no adjustments).

**Table 4:** Energy dispersive x-ray spectrometry (EDS) measurements of Ca/P and Ca/C ratios, and Mg and Na wt % in the test groups before immersion and after 20 rounds of immersion. Kruskal–Wallis test revealed no statistical significance before and after the immersion for each group at α = 0.05.

	Ca/P		Ca/C		Mg (wt %)		Na (wt %)	
	Before Immersion	After 20 Rounds	Before Immersion	After 20 Rounds	Before Immersion	After 20 Rounds	Before Immersion	After 20 Rounds
GA	1.95	1.96	10.45	8.26	1.32	1.56	2.10	2.10
GB	1.99	1.92	10.65	8.71	1.26	1.48	2.02	2.00
GC	1.97	1.98	10.21	9.13	1.50	1.41	2.09	1.83
GD	1.94	1.94	9.79	8.63	1.46	1.55	2.10	1.95
GE (control)	1.80	1.76	7.85	6.76	1.62	1.52	2.46	2.25
